# Confounding association between plasma HDL‐C levels and increased fracture risk: A correspondence

**DOI:** 10.1002/hsr2.2187

**Published:** 2024-06-20

**Authors:** Arooba Noor Abbasi, Syed Faiq Qaiser, Fatimah Hoda, Aaima Memon, Arooba Lakho

**Affiliations:** ^1^ Department of Medicine, Dow Medical College Dow University of Health Sciences Karachi Pakistan

**Keywords:** apolipoprotein A‐I, bone mineral density, cardiovascular disease, elderly, fracture, geriatrics, high density lipoprotein cholesterol, orthopaedics

## Abstract

**Background:**

This article explores the association between fractures, particularly in the elderly, and elevated plasma high‐density lipoprotein cholesterol (HDL‐C) levels. The study challenges the conventional idea of HDL‐C as “good cholesterol” by revealing its potential role as a risk factor for fractures. Factors contributing to fractures in the elderly, such as diminishing bone strength due to aging‐related tissue breakdown, are discussed. Sedentary lifestyles, low bone mineral density (BMD), and habits like smoking and alcohol consumption compound fracture susceptibility.

**Materials and Methods:**

The study delves into mechanisms linking elevated HDL‐C to fractures, using data from the ASPREE‐Fracturesub‐study of the ASPREE trial involving Australian and American participants aged 65 and above.

**Results:**

The study showed that over a 4‐year period, elevated HDL‐C levels in healthy older people were linked to a 14% higher fracture risk. This revelation expands the understanding of fracture risk factors beyond the established norms.

**Conclusion:**

The article emphasizes the need to reconsider HDL‐C's traditional role as an indicator of cardiovascular health, particularly in light of medications like Statins and Anacetrapib that raise HDL‐C levels. It calls for further exploration into the relationship between HDL‐C, fractures at varying sites, and different age groups. Practical implications involve incorporating fracture risk associated with high HDL‐C into clinical considerations, alongside advocating lifestyle changes for optimal HDL‐C levels. In summary, this study prompts a reevaluation of HDL‐C's implications in clinical practice, demanding further investigation into the intricacies of this relationship.

## 1 INTRODUCTION

The ageing process involves significant risk factors such as the gradual breakdown of tissue structure and the impairment of physiological function across a number of systems, which can manifest most noticeably in a decrease in lower limb strength. Since bones have a natural tendency to become fragile over the years, the majority of fractures occurring in individuals over the age of 60 are induced by a combination of weak bones and falling.^1^ Lack of physical activity is also a leading cause of fractures in elderly as older individuals with low bone mineral density (BMD) tend to fracture more frequently, and regular exercise has been proven to raise BMD.^2^ Additionally, smoking, a low body mass index, insufficient calcium intake, and alcohol overconsumption increase the likelihood of fractures in the elderly.^3^

### 1.1 High HDL‐C levels and fracture risk

Recently, a cohort study makes the novel finding that plasma high‐density lipoprotein cholesterol (HDL‐C) levels in healthy older persons are linked to an increased risk of fracture. As a distinctive aspect of the study, the term “healthy” refers to the absence of cardiovascular disease, dementia, physical disability, or life‐threatening chronic illnesses among the participants. Based on a post‐hoc analysis of data from the ASPREE‐Fracture sub‐study of the Aspirin in Reducing Events in the Elderly (ASPREE) clinical trial, the investigation found that a one standard deviation rise in HDL‐C levels was related to a 14% increased likelihood of fracture over a 4‐year period (HR, 1.14; 95% CI, 1.08–1.20), hence augmenting conventional risk factors for fracture.^4^ Since the Framingham study of the healthy population, HDL‐C has been termed “good cholesterol” yet recent findings have been contrary to this popular belief.^5^ That being so, the purpose of this article is to explore the association between raised plasma HDL‐C levels and increased fracture risk. We also delve deeper to identify further negative consequences on human health caused by elevated HDL‐C levels. Therefore, the message of this article is; should this association prove to be accurate, it must be applied to management strategies going forward. Although higher levels of HDL‐C have been linked with osteoporosis in a recent meta‐analysis,^6^ and preclinical studies have revealed that HDL‐C lowers bone mineral density (BMD) by decreasing the quantity and function of osteoblasts,^7^ the pathways through which an increased HDL‐C level may lead to fracture remain ambiguous. A significant possibility could be factors such as physical exercise, which may result in greater HDL‐C levels but also raise the chances of fracture. However, the validity of this explanation is weakened by the fact that fracture risk remained relatively constant when participants who practised light to vigorous workouts were omitted from the study. Additionally, pathophysiological reasoning may be indicated by a Mendelian randomisation study demonstrating a genome‐wide connection between raised HDL‐C and poor BMD.^8^ HDL‐C levels were also identified as a causative risk factor for reduced heel and lumbar spine BMD in a review of genome‐wide association studies.^9^

### 1.2 Methods employed in original study and various articles supporting this association

As part of the ASPREE study between 2010 and 2014, 16,703 Australian and 2411 American healthy older adults aged ≥70 years and ≥65 years were enlisted, respectively. The trial conducted annual checkups, during which fasting lipid levels (including HDL‐C and non HDL‐C) were measured along with anthropometric and laboratory data. Following randomisation, Australian participants in the ASPREE fracture sub‐study provided information on incident fractures. Thus, a team of specialized doctors and researchers categorized all reported fractures according to etiology and site (e.g., vertebral, hip, non‐hip). Medical imaging was utilized to diagnose the traumatic and minimal trauma fractures. Lastly, hazard ratios were computed using cox regression and an HR curve was used to analyse the potential link between HDL‐C levels and fractures.^4^ Furthermore, the evidence of a connection between HDL‐C levels and fractures is rising as a result of multiple investigations conducted globally. A cross‐sectional study carried out in the Xuhui and Yangpu districts of the Shanghai metropolitan area, China consisting of 1791 participants older than 55 years, reported a strong correlation between per standard deviation increase in HDL‐C levels and a higher risk of osteoporotic fractures in total participants and women.^10^ Likewise, according to a meta‐analysis, individuals with a lower HDL level (<40 mg/dl) had a lesser chance of bone fracture than those with a normal level (≥40 mg/dl).^11^ A 5832‐person observation from the Cardiovascular Health Study found a further positive correlation: a high cholesterol and lipoprotein profile increases the risk of hip fracture.^12^ Similar to this, a thorough investigation of the pathophysiology of elevated HDL‐C and the risk of osteoporotic fractures was conducted by comparing bone mineral density to HDL‐C, which revealed that 214 postmenopausal women had lower BMD in selected regions with high HDL‐C values.^13^ Thus, these studies highlight how elevated HDL‐C levels are associated with weaker bones and increase the risk of fracture.

### 1.3 Potential adverse effects of high HDL‐C levels

The implication of increased HDL‐C quantities being a traditional risk factor for fractures shifts attention towards additional adverse effects of extremely high plasma levels of this cholesterol. Recent studies have shown that HDL‐C is a poor indicator of the positive functions of high density lipoproteins and cannot be used to reflect the structural and functional properties of the lipids and proteins present in HDL. These factors demonstrate that HDL‐C is a poor target for medication therapy, especially in light of the consistent failure of drug trials.^14^ HDL‐C dysfunction amplifying illness was also seen in populations with pre‐existing conditions such as coronary heart or kidney disease. High levels of HDL‐C caused impairment in positive vascular effects, such as enhancing LDL‐induced monocyte chemotactic activity by cells in artery wall and increasing LDL oxidation, thus contributing to the anti‐atherogenic properties of HDL‐C.^15^ Additionally, latest studies of genetic mutations in APOA1 (apolipoprotein A‐I), a major gene of high density lipoprotein, show a direct association with increased risk of ischemic heart disease which is independent of plasma levels of HDL‐C.^16^ Encouraging current chronic disease in older adults, HDL‐C has been shown to enhance any present systematic inflammation that occurs with age.^17^ These aforementioned observations, further highlighted in Table 1, help us recognize that raising HDL‐C through drug therapy has negative consequences in healthy older adults due to its inherent damaging properties.

**Table 1:** Relevant literature summary.

Author	Investigator	Title	Source	Findings of the study
Zhou J et al. 2021	[1]	A prospective cohort study of the risk factors for new falls and fragility fractures in self‐caring elderly patients aged 80 years and over	BMC Geriatrics	Assessing elderly fall risk and fragility fractures can be effectively done using tools like the Timed Up and Go (TUG) test, walking speed, Activities of Daily Living (ADL) score, and fall risk assessment scale
Han S et al. 2020	[2]	Changes in physical activity and risk of fracture: a Korean nationwide population‐based cohort study	Scientific Reports	Consistent and frequent physical activity is linked to preventing fractures
Woolf AD et al. 2003	[3]	Preventing fractures in elderly people	BMJ	Fracture prevention strategies include reducing falls, optimizing bone strength, and targeted pharmacological treatment for highest risk individuals Strong risk factors for future fractures include low bone density and previous fractures; they can be combined with additional risk variables to identify the population most at risk.
Hussain SM et al. 2023	[4]	Association of Plasma High‐Density Lipoprotein Cholesterol Level With Risk of Fractures in Healthy Older Adults	JAMA Cardiology	Elevated levels of HDL‐C are linked to a higher likelihood of experiencing fractures. This association was independent of common risk factors for fractures.
Gordon DJ et al. 1989	[5]	High‐density lipoprotein cholesterol and cardiovascular disease. Four prospective American studies	Circulation	Epidemiological data suggest an independent inverse association between HDL‐C levels and CHD event rates, with a 2%–3% decrease in CHD risk for each 1‐mg/dl increase in HDL‐C levels. While increasing HDL‐C levels may enhance the benefits of lowering LDL‐C levels, the efficacy of therapies aimed specifically at increasing HDL‐C remains uncertain, prompting a focus on adopting healthy lifestyle measures like exercise, weight loss, and smoking cessation.
Zhao H et al. 2021	[6]	Blood lipid levels in patients with osteopenia and osteoporosis: a systematic review and meta‐analysis	Journal of bone and mineral metabolism	Patients with osteoporosis exhibited elevated levels of HDL‐C.
Papachristou NI et al. 2017	[7]	High‐Density Lipoprotein (HDL) metabolism and bone mass	*Journal of Endocrinology*	Recent literature emphasizes a direct link between dysfunctional HDL and metabolic disorders, including bone diseases, emphasizing the need to address key regulatory factors and understand the role of bone marrow adiposity. Investigating the structure‐function relationship of HDL beyond HDL‐C levels is crucial for developing effective therapies targeting HDL functionality in bone pathologies.
Chen H et al. 2020	[8]	Are blood lipids risk factors for fracture? Integrative evidence from instrumental variable causal inference and mediation analysis using genetic data	Bone	There is a causal relationship between HDL and fractures, but it does not provide substantial direct evidence for causal links between other lipids and fractures. Additionally, HDL and triglycerides (TG) might indirectly impact fractures through their effects on bone mineral density (BMD).
Zhang Q et al. 2021	[9]	Detecting causal relationship between metabolic traits and osteoporosis using multivariable Mendelian randomization	Osteoporosis International	By combining MR techniques, The probable causative risk factors for FN, FA, LS, and heel BMD were identified individually. The potential causal risk factors for BMD were also prioritized and ranked.
Wang Y et al. 2018	[10]	Association between Serum Cholesterol Level and Osteoporotic Fractures	Frontiers in Endocrinology	In Chinese elderly individuals, Serum HDL‐C level is strongly correlated with a risk of osteoporotic fractures in women, while serum TG level is significantly correlated with a risk of osteoporotic fractures overall and specifically among women with TG levels exceeding 1.64 mmol/L.
Ghorabi S et al. 2019	[11]	Lipid Profile and Risk of Bone Fracture: A Systematic Review and Meta‐Analysis of Observational Studies	Endocrine Research	Higher plasma levels of total cholesterol were correlated with an elevated incidence of bone fractures. Furthermore, lower levels of HDL were linked to a heightened risk of osteoporotic fractures
Barzilay JI et al. 2022	[12]	The association of lipids and lipoproteins with hip fracture risk: the cardiovascular health study	The American Journal of Medicine	In older persons, there is a correlation between hip fracture risk and lipoproteins and lipids. The relationships are intricate. Mechanistic investigations are required to comprehend these results.
Yamaguchi T et al. 2002	[13]	Plasma lipids and osteoporosis in postmenopausal women	Endocrine Journal	Plasma LDL‐C and HDL‐C levels were favorably and inversely correlated with both R‐ and L‐BMD values, respectively. while low plasma TG levels were linked to the prevalence of vertebral fractures in postmenopausal women, Thus, plasma lipids may be the common component underlying both osteoporosis and atherosclerosis and may be connected to both bone mass and bone fragility.
Annema W et al. 2016	[14]	Dysfunctional high‐density lipoproteins in coronary heart disease: implications for diagnostics and therapy	Translational Research	Due to HDL's intricate structural and functional makeup, drugs intended to prevent or treat cardiovascular disorders have not been able to effectively target it.
Besler C et al. 2012	[15]	Molecular mechanisms of vascular effects of High‐density lipoprotein: alterations in cardiovascular disease	EMBO Molecular Medicine	Low levels of HDL cholesterol increase the risk of coronary disease and myocardial infarction, challenging the traditional notion of HDL as purely anti‐atherogenic reliable assays are needed to assess HDL functionality accurately. HDL‐raising therapies with vasoprotective properties to effectively reduce cardiovascular risk should be prioritized.
Frikke‐Schmidt R. 2010	[16]	Genetic variation in the *ABCA1* gene, HDL cholesterol, and risk of ischemic heart disease in the general population	Atherosclerosis	Recent ABCA1 studies in the general population reveal a genetic mixture of rare alleles with large effects and common alleles with modest effects on common traits. While both common and rare ABCA1 variants influence HDL cholesterol levels and ischemic heart disease (IHD) risk, the association between ABCA1 variants and IHD risk appears independent of plasma HDL cholesterol levels, challenging the causality of isolated low HDL cholesterol in IHD development.
McTaggart F et al. 2008	[17]	Effects of statins on high‐density lipoproteins: a potential contribution to cardiovascular benefit.	Cardiovascular Drugs and Therapy	Statins induce slight elevations in HDL‐C and apo A‐I, likely through reductions in CETP activity. It is conceivable that these alterations independently contribute to the cardiovascular advantages associated with statins, although additional research is required to delve deeper into this potential connection.
Bowman L et al. 2017	[18]	Effects of anacetrapib in patients with atherosclerotic vascular disease.	The New England Journal of Medicine	In patients with atherosclerotic vascular disease receiving intensive statin therapy, the use of anacetrapib led to a reduced occurrence of major coronary events compared to those receiving a placebo.
Bergman GJD et al. 2010	[19]	Efficacy of vitamin D3 supplementation in preventing fractures in elderly women: a meta‐analysis	Current Medical Research & Opinion	Vitamin D3 at a dosage of 800 IU per day can help lower the risk of osteoporotic non‐vertebral, hip, and non‐vertebral non‐hip fractures in older women. For non‐vertebral and non‐vertebral‐non‐hip fractures, vitamin D3 and calcium supplementation seems to provide advantages above calcium supplementation alone.
März W et al. 2017	[20]	HDL cholesterol: reappraisal of its clinical relevance.	Clinical Research in Cardiology	HDL‐C correlates with cardiovascular risk solely in healthy individuals. its low levels warrant examination for metabolic and inflammatory issues. It is beneficial and advised to raise HDL‐C through lifestyle modifications including quitting smoking and exercising. HDL‐C is not a viable target for medication currently.
Singh IM et al. 2007	[21]	High‐density lipoprotein as a therapeutic target. A systematic review	The Journal of the American Medical Association	There is limited evidence supporting the aggressive increase of HDL‐C levels beyond what lifestyle changes achieve alone. Ongoing clinical trials focusing on specific pathways in HDL metabolism may offer new options for cardiovascular treatment.

## 2 CONCLUSION

The results of the cohort study under discussion^4^ are relevant in clinical practice, particularly in light of the adverse effects of atheroprotective medications that significantly raise plasma HDL‐C quantities. Notable drugs such as Statins^17^ and Anacetrapib^18^ reduce the risk of cardiovascular disease, primarily by lowering LDL aided by the ability to also increase HDL‐C. The pathways through which HDL‐C (in physical activity or drugs such as statins) is related to a greater risk of fracture remain unclear,^4^ and further study is required to identify the quality of HDL and the molecular basis underlying this association. Nevertheless, clinicians must take into account the detrimental effects of high HDL‐C levels when considering the potential advantages of these medications, particularly for the elderly. Given that an average level of 89 mg/dl of HDL‐C (the highest quintile in the cohort study) has been associated with a 33% higher risk of fracture,^4^ it is evident that measuring HDL‐C is applicable to people at risk of fracture. However, additional investigation is required to guarantee its reliability and necessity. Moreover, fracture prevention in the elderly population has conventionally included Vitamin D3 and calcium supplementation.^19^ To avoid fractures, maintaining normal HDL‐C levels must also be crucial in clinical practice. Ideally, the recommended strategy for raising HDL‐C levels within a healthy range is through lifestyle modification.^20^ Incorporating a diet rich in unsaturated fatty acids, smoking cessation, aerobic exercise, and weight loss are essential lifestyle changes that have been reported to raise HDL‐C.^21^ To include the consequences of high plasma HDL‐C levels in future management plans, further research is required to ascertain whether there is an increased tendency for fractures to develop at specific sites (e.g., hip or vertebrae) and if this association holds true for people of all age groups.

The clinical findings about fractures, their prevention, and their relationship to HDL‐C are mentioned in this table. It also delves into the various adverse impacts of HDL‐C that have been discovered over time. The results were reached following a thorough literature review, with citations listed in the aforementioned table.

## AUTHOR CONTRIBUTIONS

**Arooba Noor Abbasi**: Conceptualization; Writing—original draft; Writing—review & editing; Investigation. **Syed Faiq Qaiser**: Conceptualization; Writing—original draft; Writing—review & editing; Investigation. **Fatimah Hoda**: Conceptualization; Writing—original draft; Writing—review & editing; Investigation. **Aaima Memon**: Conceptualization; Writing—original draft; Writing—review & editing; Investigation. **Arooba Lakho**: Conceptualization; Writing—original draft; Writing—review & editing; Investigation.

## ETHICS STATEMENT

None (our work does not involve live subjects, human or animal; therefore there was no need of an ethics approval).

## TRANSPARENCY STATEMENT

The lead author Arooba Noor Abbasi affirms that this manuscript is an honest, accurate, and transparent account of the study being reported; that no important aspects of the study have been omitted; and that any discrepancies from the study as planned (and, if relevant, registered) have been explained.

## Data Availability

Data sharing not applicable to this article as no datasets were generated or analysed during the current study. All authors have read and approved the final version of the manuscript.
